# From Fishing to Fish Processing: Separation of Fish from Crustaceans in the Norway Lobster-Directed Multispecies Trawl Fishery Improves Seafood Quality

**DOI:** 10.1371/journal.pone.0140864

**Published:** 2015-11-16

**Authors:** Junita D. Karlsen, Ludvig Ahm Krag, Christoffer Moesgaard Albertsen, Rikke Petri Frandsen

**Affiliations:** 1 DTU Aqua, National Institute of Aquatic Resources, North Sea Science Park, DK-9850, Hirtshals, Denmark; 2 DTU Aqua, National Institute of Aquatic Resources, Jægersborg Allé 1, DK-2920, Charlottenlund, Denmark; Aristotle University of Thessaloniki, GREECE

## Abstract

Fishing gears have negative impacts on seafood quality, especially on fish in the mixed trawl fishery targeting Norway lobster (*Nephrops norvegicus*). In this fishery, which is worth about €80 millions in Denmark alone, the quality of fish can be significantly improved by simple gear changes. A trawl codend divided into an upper and lower codend was designed to separate fish from Norway lobster during the fishing process by encourage fish to swim into the upper codend by using a frame at the entrance of the lower codend. Separate codends for fish and Norway lobster in the same gear provide the opportunity to selectively reduce small low-value fish, which will reduce catch weight and sorting time onboard the vessel. For this horizontally divided test codend and a standard codend, in which the catch was mixed, quality assessments were performed on the same batches of fish during three steps of the value chain: i) aboard the fishing vessel; ii) at the Fishermen’s Collection Central, and iii) in the production plant. Four species of fish and fillets from fish caught in the upper codend of the test codend were of significantly better quality for several of the assessed parameters compared with those caught in the standard codend: i) newly caught fish showed significantly less scale loss and discolourations and had significantly better texture; ii) landed fish had significantly better skin appearance and texture and significantly fewer discolourations; and iii) fillets showed significantly fewer blood spots and had significantly better texture. There were no differences in injuries for newly caught fish or gaping and bruises for fillets between the test and standard codends. The decrease in catch-related damages in the test codend is explained by little contact between fish and animals with hard or spiny surfaces due to successful separation of fish and Norway lobster into the upper and lower codends, respectively, and by lower catch weight in the upper codend of the test codend compared with the standard codend. The decrease in damages may also improve quality indirectly by inflicting less stress to the fish and subsequently give better texture, which offers advantages such as pre-rigor filleting and fresher products for the market. Significant improvements in fish quality can potentially increase the catch value in nationally important fisheries.

## Introduction

On its way through the value chain from fishing vessel to consumers, seafood quality is assessed based on its freshness at several stages. Danish fishermen usually deliver their catch to Fishermen’s Collection Centrals, which label the catch according to the freshness categories Extra, A, and B (defined by EU regulations) prior to the fish auction [1]. This freshness assessment involves visual inspection of the condition of the body surface of fish and other seafood, including pressure marks, discolouration, and injuries. Subsequently, exporters at the fish auctions evaluate freshness of the products based on their personal experience from handling raw materials. This assessment, together with the product portfolio and the current market situation, influence the price the companies are willing to pay for the displayed products. For consumers, for whom the catch date is unknown, evaluation of species-specific freshness when buying fresh seafood is facilitated by apps on smartphones and tablets. Such apps are based on properties of the seafood that change during the storage period, such as appearance, odour, and texture [2, 3]. Freshness is thus associated with both the degree of spoilage and damage to the seafood.

Seafood quality is at its best in the first step of the value chain, as reduced quality at this point obviously cannot be improved at a later stage. Fishing gears affect fish quality in several ways, and some fishing methods have more profound impacts than others [4–7]. Rotabakk et al. [8] found that several quality parameters were lower (e.g., more bruises, less firm texture, poor bleeding, lower pH, lower sensory quality) for Atlantic cod (*Gadus morhua*) caught in trawl fisheries compared to those caught by longlining. The proportions of trawl-caught fish that suffer injuries are high (50–95%) and increase with catch size for catches < 5 tonnes [7–10]. Focus on increased fish quality can be valuable for the fishing industry, especially in areas where there are quota-restrictions for most commercially targeted fish and crustaceans [7, 9, 11, 12].

The mixed trawl fishery targeting Norway lobster (*Nephrops norvegicus*) is one of the most economically important European fisheries. In addition to Norway lobster several species of fish are also targeted and constitute a substantial fraction of the total catch value. This fish catch is known among fishermen and exporting companies as ‘lobster fish’, which refers to the relatively low perceived quality compared to trawl fisheries targeting fish only. Trawls targeting Norway lobster are in close contact with the seabed, and debris and benthic organisms with hard and spiny outer surfaces are caught alongside Norway lobster and fish. As this mixed catch builds up in the aft end of the trawl (i.e., the codend) scales may be scraped off the fish skin and lead to injuries during several steps of the fishing process. First, codend pulsing due to vessel motion and water turbulence in the aft end of the codend causes displacement of the catch during towing [13, 14]. This may increase the contact between spiny invertebrates and fish. Second, at the end of the haul the trawl is lifted from the sea bed to the sea surface. Waves acting on the codend cause further back-and-forth movements of the catch, which becomes compressed and crowded in the aft part of the net [15, 16]. Fish that cannot avoid contact with the netting or other individuals in the net tend to panic. Vigorous swimming in all directions further increases the contact rate with the netting and thus the risk of abrasions [17, 18]. Third, when the codend is lifted aboard the vessel, the fish are pressed against other catch components and the netting, which can result in bruises and pressure marks on the skin and muscle [8, 19, 20]. Finally, in the holding bin fish are pressed against other catch components. In addition to physical damage, stress due to exhaustion affects fish texture negatively by lowering muscle pH and accelerating the onset and resolution of *rigor mortis* [21–24]. This in turn can result in more gaping of fillets [25, 26]. ‘Lobster fish’ are thus susceptible to quality downgrading and are therefore more likely to be used in low-value products.

Separating fish from Norway lobster and other organisms with hard outer surfaces in the fishing gear has the potential to improve several quality parameters compared with gears in which catch components are mixed. Gears separated into two horizontal compartments in the trawl mouth and which lead to two separate codends can efficiently sort the catch during the fishing process. Roundfish such as haddock (*Melanogrammus aeglefinus*), saithe (*Pollachius virens*), and whiting (*Merlangius merlangus*) are mainly caught in the upper codend of the gear, whereas cod, flatfish, and Norway lobster are mainly caught in the lower codend [27–29]. Designs in which the separation is located in the aft end of the trawl can separate most Norway lobster from the majority of fish, including cod and flatfish [30, 31]. Main & Sangster [27] observed that bruises and scale loss of fish caught in the lower codend of their separated gear, in which they also caught crustaceans and other benthic organisms, weeds, and stones, were absent in fish caught in the upper codend, where mostly fish were caught. Furthermore, separate codends for fish and Norway lobster provide the opportunity to selectively reduce small low-value fish, which will reduce catch weight and sorting time [32]. Decreasing the pressure present when lifting the catch onboard the vessel and in the holding bin potentially can increase the product value of the retained catch by increasing fillet yield through fewer bruises and less discolouration [11].

If the amount of damages in the catch can be reduced by altering fishing gear designs, there is also potential of obtaining higher product prices [11]. This potential of increasing the value of the quota is an important aspect when fishermen consider whether to implement a new gear design or not. The effect of quality on the price is however not straightforward to document due to the complex set of other factors influencing price at the fish auction including market demand for whole versus processed fish, product batch sizes, and if the fish is sold early or late during the auction. Nevertheless, one prerequisite for obtaining higher prices is that the buyers are able to perceive the quality improvements of the landed fish. Quality assessments of catch from new gear designs should therefore include quality parameters that are essential for exporters. Subjective sensory evaluations are important throughout the value chain, but to allow for a quantitative comparison of quality variations related to different gear designs, a systematic and standard registration of damages is necessary. Scoring schemes describe the type and degree of damages and indicate what score to give damages of different severity. This method reduces the subjectivity of panel members during assessment, and has previously been used to develop a Catch-damage-index and to assess the effect on gear changes on quality of fish [7, 20].

The main objective of this study was to investigate whether the quality of fish caught in the mixed trawl fisheries targeting Norway lobster can be significantly improved by simple gear changes. A codend designed to separate fish and Norway lobster into an upper and lower codend, respectively, was tested during commercial fishery and compared to a standard codend in which the catch were mixed. The goal was to obtain results that potentially could increase the revenue of the industry. Exporters were thus included in the quality assessment, which consisted of sensory analyses of parameters important to them when buying fish at the fish auction.

## Materials and Methods

### Ethics statement

This study did not involve endangered or protected species. Experimental fishing was conducted onboard a Danish commercial trawler in accordance with the fishing permit granted by the Danish AgriFish Agency (Ref. no. 13-7410-000243) and the Norwegian Directorate of Fisheries (Ref. no. 13/12085). No further permits were required to conduct the study. The fish was handled according to commercial practises and were landed and sold for human consumption after the quality assessments.

### Gear design, fishing operation, and handling

**Fig 1:**
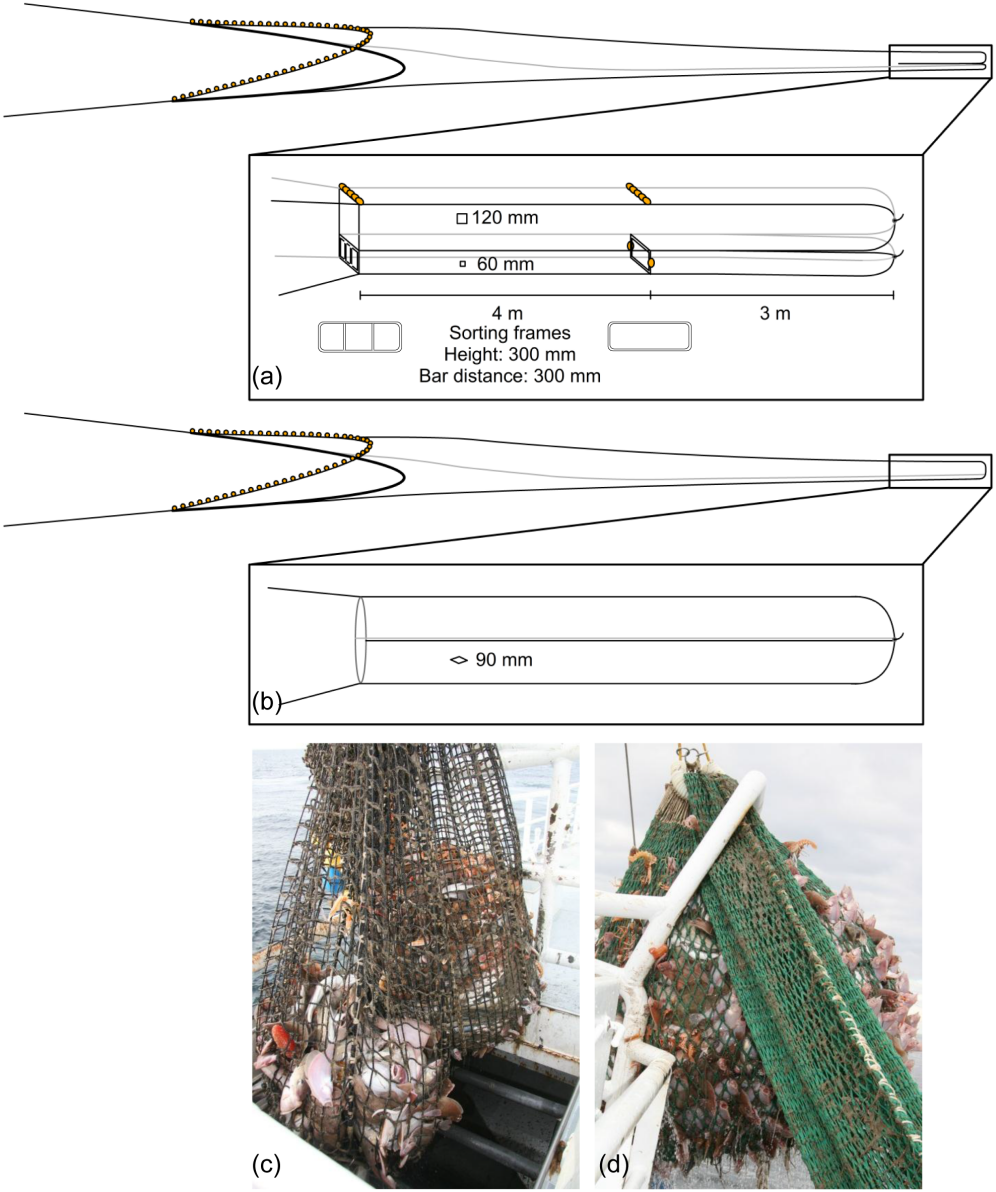
Schematic illustrations of (a) the vertically divided fishing gear showing the upper and lower codends and the sizes of their square meshes in addition to the two frames used to keep the lower codend open, and (b) the standard codend showing the size of the diamond meshes. Photos of (c) the upper and lower codends just before the catch of the upper codend is emptied into the holding bin of the vessel, and (d) the standard codend as the catch is hauled up on deck.

The test codend was developed to separate fish from Norway lobster by exploiting differences in their behaviour. It was made of four net panels and was divided into an upper and a lower codend by a horizontal net panel. In the aft end the separation continued into two split codends. The upper and lower compartment and codend are herafter referred to as the upper and lower codend, respectively. The upper codend was intended for fish, which tend to keep in the largest passage towards the catch accumulation and have a relatively good swimming capacity compared with Norway lobster [31]. At the entrance of the upper codend the side panels were about 60 cm high and the cross section was designed to constitute the upper two-thirds (Fig 1). The side panels at the entrance of the lower codend were about 30 cm high and were designed to constitute the remaining one-third of the cross section. The lower codend was intended for Norway lobster, which moves passively along the bottom of the gear [33]. A reduction in body damages depended on successful separation of fish and Norway lobsters, thus two frames (90 cm x 30 cm) made from 20 mm stainless steel pipes were mounted in the lower codend to keep it open so Norway lobster could enter (Fig 1). Also, the foremost frame had two vertical bars placed 30 cm apart to create a visual obstacle to encourage fish to swim into the upper codend. To further optimise catch separation, the foremost frame was placed in the transition between the tapered and non-tapered section of the gear where the inclination of the lower netting of the trawl ends (Fig 1). The test codend was also designed to allow undersized fish and Norway lobster to escape; the upper and lower codends consisted of Ultra Cross netting made of 120 mm and 60 mm square meshes, respectively. The catch in each of these two codends was compared to that in the standard codend, which was made of 90 mm diamond mesh double 4 mm PET-netting commonly used in commercial codends. In the standard codend all catch components were mixed following commercial practice.

The experiment was conducted aboard a commercial trawler (162 BT, 22 m, 299 kW) using a three wire towing rig so that the test codend was fished side by side with the standard codend during all hauls. Fishing was conducted during the day from 24 September to 2 October 2013 on commercial fishing grounds in Skagerrak. The geometry of the entrance of the test codend was monitored by underwater video recordings. The catch was hauled on deck on the port side of the vessel and emptied into a holding bin. The catches from the three codends were kept separate during handling and quality assessment. The handling sequence of the different codends changed between hauls to avoid systematic influence on the quality assessment related to the amount of time the fish were kept in the codend. The catch was taken from the holding bin, gutted by hand, washed, and sorted by species and size prior to the quality assessment. Subsequently, the catch was stored on ice at 2°C in the vessel’s cold store. Catch from the conducted hauls was landed at the Fishermen’s Collection Central twice during the field period; six days after the cruise started and again after nine days when the cruise ended.

### Quality assessments

**Fig 2:**
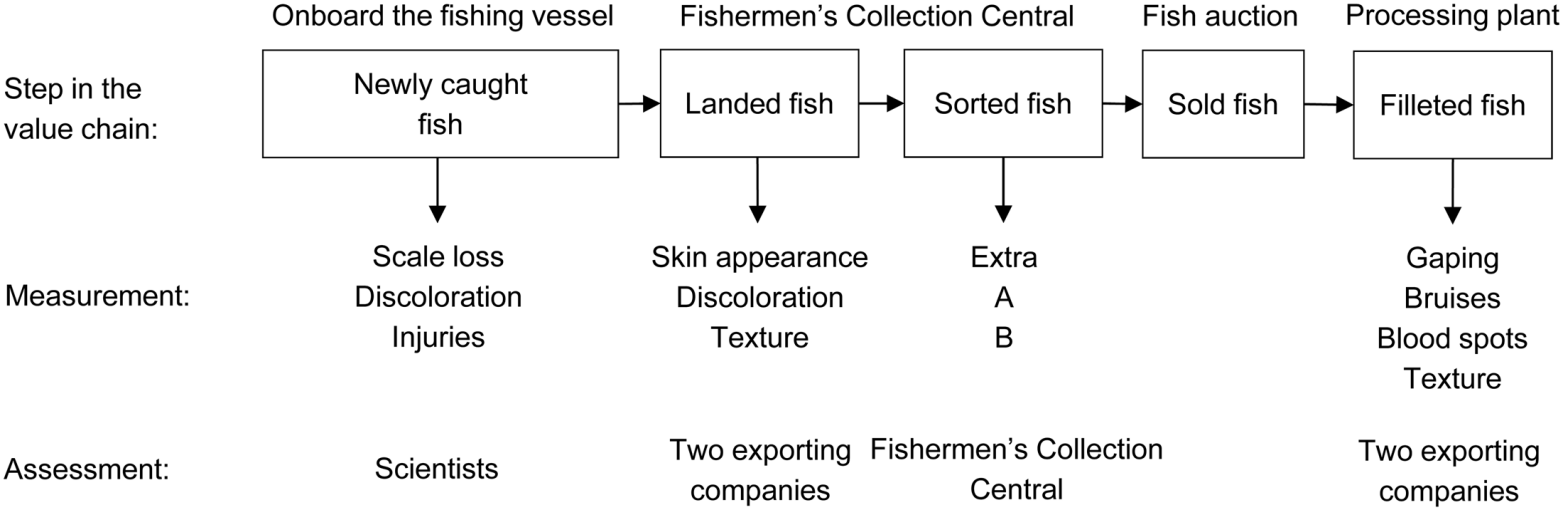
An overview of the data collection and assessments during the different steps of the value chain.

Quality of fish was assessed during three steps of the value chain, as described below. Visual and tactile analyses conducted by panels of scientists and exporters were used in the quality assessment and a score was given according to a defined scoring scheme to indicate the severity of each type of damage. Fig 2 provides an overview of the different quality parameters included in each assessment. All samples were marked and tracked through all steps in the value chain using colour codes.

#### Newly caught fish

Samples of cod, saithe, plaice (*Pleuronectes platessa*), witch flounder (*Glyptocephalus cynoglossus*) from the three codends were randomly collected from the processing line and assessed by two scientists aboard the fishing vessel (Fig 2). After visual inspection, individual fish was given a score (0–2) for each of the quality parameters scale loss, discolouration according to the point system described in Table 1. A lower score indicates better quality.

**Table 1:** Description of the assessed damages found in fish and the scoring scheme according to the severity of the damage.

	Damage	Description	Score		
			0	1	2
*Newly caught fish*	Scale loss	Areas with no/few scales. Abrasions due to contact with invertebrates etc. or the netting.	No	NA, percentage of body surface without scales was estimated	NA, percentage of body surface without scales was estimated
	Discolouration	Bruises, areas of blood extravasations visible on the skin, probably due to squeezing the fish. Blood spots, small spots, probably caused by contact with spiny objects.	No	Few, weak	Many or severe
	Injuries	Ruptures/tears in the skin, probably caused by the netting or contact with invertebrates etc.	No	Few, small punctures/holes in skin	Many or skin tears/crushed muscle tissue
*Landed fish*	Skin appearance	Colour, presence and consistency of mucus, degree of scale loss.	Glossy, no/little scale loss	Bright, but without shine/rather dull, small areas of scale loss	Lacklustre/dull, large areas of scale loss
	Discolouration	Bruises, areas of blood extravasations visible on the skin, probably due to squeezing the fish. Blood spots, small spots, probably caused by contact with spiny objects.	No	Few, weak	Many, severe
	Texture	Stiffness of the fish	Pre-rigor/rigor	Firm	Soft
*Fillet*	Gaping	The visual appearance of the fillet surface, deepness and extent of slits in the fillet.	Smooth	Few, slight gapings	Deep/many gapings
	Blood spots	Small blood extravasations visible on the skin, probably due to surface contact between fish and spiny objects.	No	Few, weak	Many, severe
	Bruises	Areas of blood extravasations visible on the fillet, probably due to squeezing of the fish.	No	Small, weak	Large/many, severe
	Texture	Stiffness of the fillet.	Pre-rigor/rigor	Firm	Soft

#### Landed fish

Once the fish were landed, the quality assessment of samples of cod, saithe, plaice, and witch flounder was conducted by a panel consisting of a person from the assessment staff of each of two exporters (Strandby Fiskeeksport ApS and Rasmus Clausen & Sønner ApS) that buy and process fish from the fish auction daily (Fig 2). These two persons participated throughout the study. This assessment was performed as a blind test in which the panel members were presented with batches of fish without knowing from which codend the catch originated. Based on visual and tactile inspection of fish, the assessment panel gave scores (0–2) for skin appearance, discolouration, and texture of fish from the three different codends separately for each catch day (Table 1). These quality parameters are the ones most important to the exporting companies when they evaluate fish prior to bidding on it at the fish auction. A mean score was given for the whole sample, and it reflected the overall assessment of the given species caught in the given codend for a given catch day (haul).

After the quality assessment, the sampled fish were sorted by the Fishermen’s Collection Central into size classes and into the EU freshness categories Extra, A, and B while maintaining traceability to catch date and codend (Fig 2). According to EU regulations, fish in freshness category Extra must be free of pressure marks, injuries, blemishes, and bad discolouration [1]. In freshness category A, a very small proportion of the fish can have slight pressure marks and superficial injuries, whereas in freshness category B a small proportion with more serious pressure marks and superficial injuries is tolerated.

#### Fillets

Approximately 4–6 hours after the fish auction, fillets were evaluated by the same panel that assessed the landed fish in their processing plant (Fig 2). Samples of 5–10 skin and boneless fillets from cod, saithe, plaice, and witch flounder were taken from every second catch day. Scores (0–2) were given for gaping, blood spots, bruises, and texture (Table 1). The exporters considered these attributes of the product to be the most important. The total score for each species and attribute was used to compare the relative quality of the fish from the three different codends.

### Analyses of quality assessments

All of the different quality assessments where analysed individually using a cumulative logit model with proportional odds ([34], see S1 Appendix for details). For newly caught fish the models included codend and species, with interaction, as covariates and haul as a random effect to accommodate for differences in catch levels and composition of the catch between hauls. The models for landed fish and fillets included codend, species, and time since catch as covariates and haul as a random effect. No interactions were included to prevent overfitting the data. For discolouration and scale loss, additional analyses were performed to relate the quality assessments to the total catch weight and proportion of Norway lobster, respectively. The model for discolouration also included codend and species, with no interactions, whereas the model for scale loss included species and the interaction between proportion of Norway lobster and species. The latter analysis was conducted for each codend separately. A proportional odds model was also used to relate the quality assessments of the two exporting companies with quality classes from the Fishermen’s Collection Central by including the quality assessments as explanatory variables along with species, days since capture, and a random effect on the observation date.

All models were reduced as much as possible using likelihood ratio tests [35]. Inference was performed in R using the package TMB [36]. When the value from the calculation was 1, there was no difference between the test and standard codend. When the value was > 1 the quality in the test codend was better than that in the standard codend, and the opposite was true when the value was < 1. The quality in the codends was significantly different when 1 was not included in the confidence interval. p-values for individual parameters were calculated using the Wald test.

## Results

### Fishing gear and fishing operation

**Fig 3:**
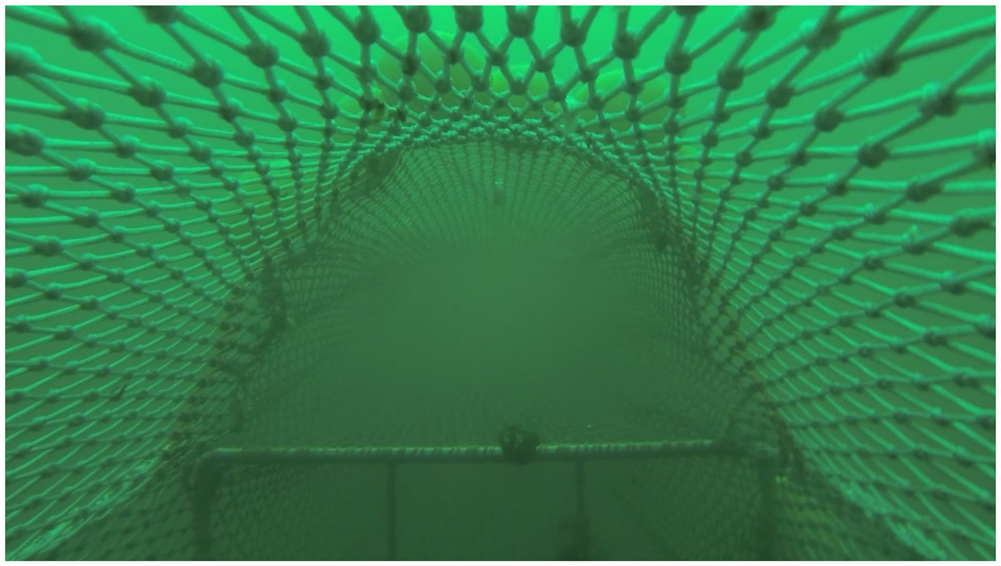
Photo of the test codend showing the frame with vertical bars mounted at the entrance of the lower codend and the geometry at the entrance of the upper codend during fishery.

Nine valid hauls were conducted during good fishing conditions (Table 2). The duration of the hauls varied between 2.5 and 6.5 hrs (mean: 5.3 hrs), which resembles commercial towing times. Underwater video recordings showed an open and stable geometry at the codend entrance of the test gear during fishing (Fig 3). There were no problems in handling the two frames in the lower codend during the fishing process and the two vertical bars in the foremost frame did not collect any objects. Except for two hauls, the catch weight in the upper codend was smaller (mean: 181 kg) than that in the lower codend (mean: 360 kg) (Table 2). In seven of the nine hauls, the total catch weight in the test codend (upper + lower) was smaller than that in the standard codend (mean: 664 kg). Of the total catch, the largest proportion of Norway lobster was caught in the lower codend (an average of 15% in a haul), and the smallest proportion was caught in the upper codend (an average of 0.3% in a haul). In the standard codend an average of 8% of the total catch consisted of Norway lobster.

**Table 2:** Operational conditions and catch weights.

Haul no.	Duration (tt:mm)	Wind direction	Wind speed (m/sec)	Sea state (m)	Sailing speed (knot)	Fishing depth (m)	Catch weight (kg)		
							Upper codend	Lower codend	Standard codend
1	04:30	N	5	0–1	2.6	104	313	273	818
2	02:25	N	4	0–0.5	2.6	113	114	123	401
3	05:45	N	5	0–0.5	2.6	122	150	315	807
4	05:30	NV	4	0–0.5	2.6	132	75	389	588
5	05:25	NØ	-	0–0.5	2.6	132	200	644	824
6	06:30	Ø	3	0–0.5	2.6	75	387	220	751
7	05:50	Ø	3	0–0.5	2.6	141	235	582	748
8	06:15	Ø	5	0–0.5	2.6	113	94	483	759
9	05:50	SØ	8	0–1	2.6	198	60	215	277

### Quality assessment

Based on the comparisons of fish caught in the upper and lower codends with those caught in the standard codend, fish caught in the upper codend had the best quality in most cases (Table 3). In the remaining cases, the quality of fish from the upper codend was similar to that of fish from the two other codends. The quality of fish from the lower and standard codends was similar.

**Table 3:** Mean scores of the quality parameters for four fish species during three steps in the value chain. Statistical comparisons were made using a proportional odds model, and significance level was calculated using the Wald test.

Step in value chain	Quality measure	Species	Mean scores (n)		
			Upper codend	Lower codend	Standard codend
Newly caught fish	Scale loss	Cod	0.47 (530)**	0.75 (406)**	0.69 (851)
		Saithe	1.00 (77)**	1.10 (134)**	1.13 (573)
		Plaice	0.50 (399)**	0.69 (378)	0.73 (561)
		Witch flounder	1.39 (119)**	1.75 (233)*	1.81 (427)
	Discolouration	Cod	0.61 (530)**	0.82 (406)	0.77 (851)
		Saithe	0.06 (77)**	0.38 (134)	0.23 (573)
		Plaice	0.80 (399)**	1.00 (378)**	1.12 (561)
		Witch flounder	0.90 (119)	0.92 (233)	0.99 (427)
	Injuries	Cod	0.03 (530)	0.04 (406)	0.04 (851)
		Saithe	0.01 (77)	0.03 (134)	0.01 (573)
		Plaice	0.03 (399)	0.02 (378)	0.04 (561)
		Witch flounder	0.03 (119)	0.03 (233)	0.02 (427)
Landed fish	Skin appearance	Cod	0.00 (9)	1.89 (9)	0.78 (9)
		Saithe	0.50 (4)	1.50 (4)	1.75 (4)
		Plaice	0.00 (6)	1.00 (6)	1.17 (6)
		Witch flounder	0.20 (5)	1.40 (5)	1.20 (5)
		Pooled	**	*	
	Discolouration	Cod	0.44 (9)	1.22 (9)	0.78 (9)
		Saithe	0.00 (4)	0.25 (4)	0.75 (4)
		Plaice	0.17 (6)	1.17 (6)	1.67 (6)
		Witch flounder	0.60 (5)	1.60 (5)	1.40 (5)
		Pooled	**		
	Texture	Cod	0.89 (9)	1.22 (9)	1.22 (9)
		Saithe	1.00 (4)	1.50 (4)	1.50 (4)
		Plaice	0.67 (6)	1.33 (6)	1.17 (6)
		Witch flounder	0.80 (5)	1.20 (5)	1.00 (5)
		Pooled	**		
Fillets	Gaping	Cod	0.90 (20)	1.10 (20)	0.65 (20)
		Saithe	1.50 (4)	0.73 (15)	0.87 (15)
		Plaice	0.27 (30)	0.13 (30)	0.33 (30)
		Witch flounder	1.13 (30)	0.63 (30)	0.83 (30)
	Bruises	Cod	0.40 (20)	0.00 (20)	0.30 (20)
		Saithe	1.00 (4)	0.10 (15)	0.60 (15)
		Plaice	0.33 (30)	0.73 (30)	0.43 (30)
		Witch flounder	0.17 (30)	0.13 (30)	0.10 (30)
	Blood spots	Cod	0.30 (20)	0.60 (20)	0.50 (20)
		Saithe	0.00 (4)	0.53 (15)	0.33 (15)
		Plaice	0.20 (30)	0.07 (30)	0.17 (30)
		Witch flounder	0.10 (30)	0.37 (30)	0.50 (30)
		Pooled	*		
	Texture	Cod	0.25 (20)	0.25 (20)	0.75 (20)
		Saithe	0.00 (4)	0.87 (15)	0.33 (15)
		Plaice	0.33 (30)	0.47 (30)	0.37 (30)
		Witch flounder	0.33 (30)	0.33 (30)	0.33 (30)
		Pooled	*		

n is the number of individuals assessed.

*p < 0.05,

**p < 0.01

#### Newly caught fish

**Fig 4:**
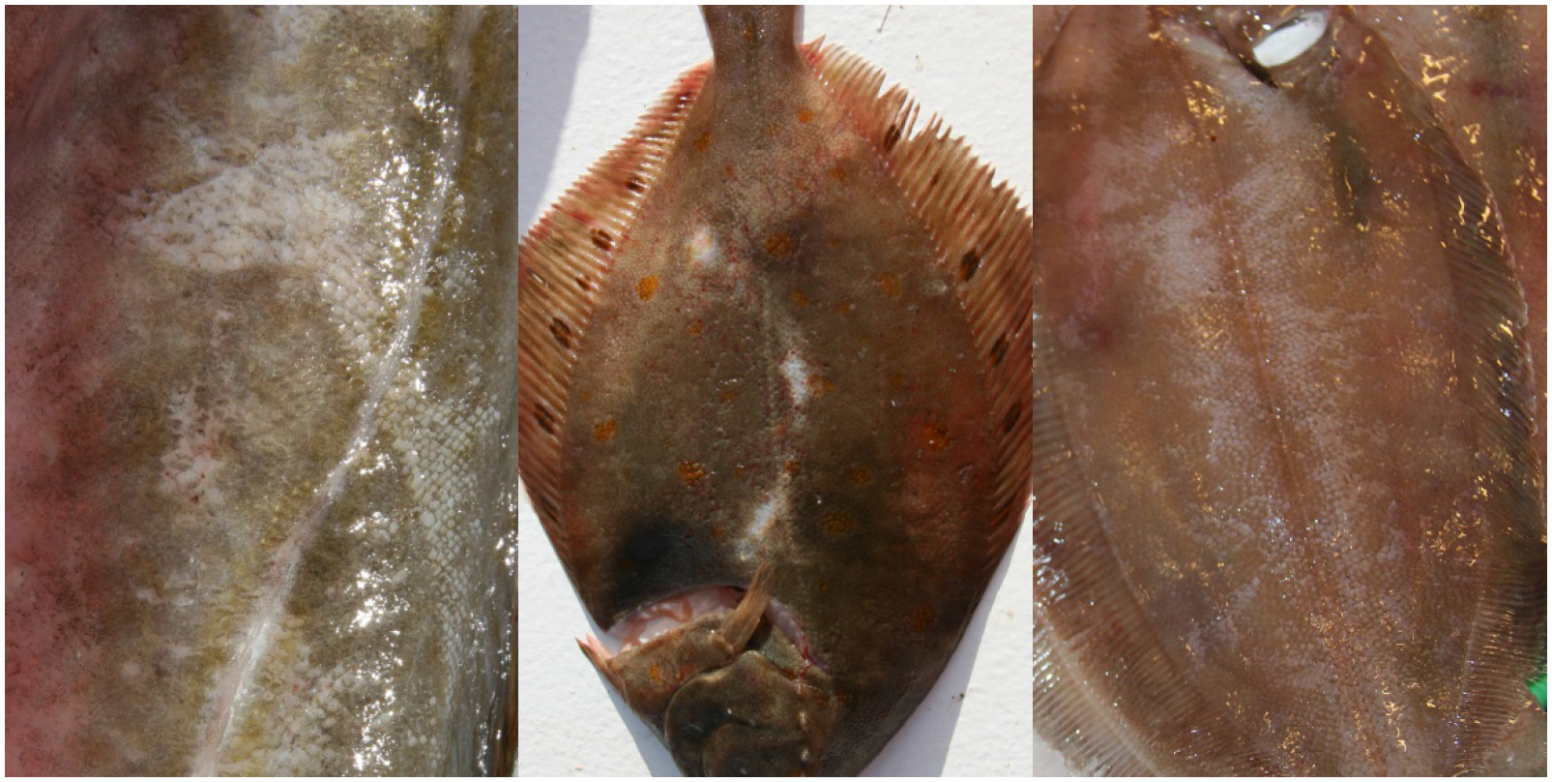
Scale loss in cod (left), plaice (middle), and witch flounder (right).

**Fig 5:**
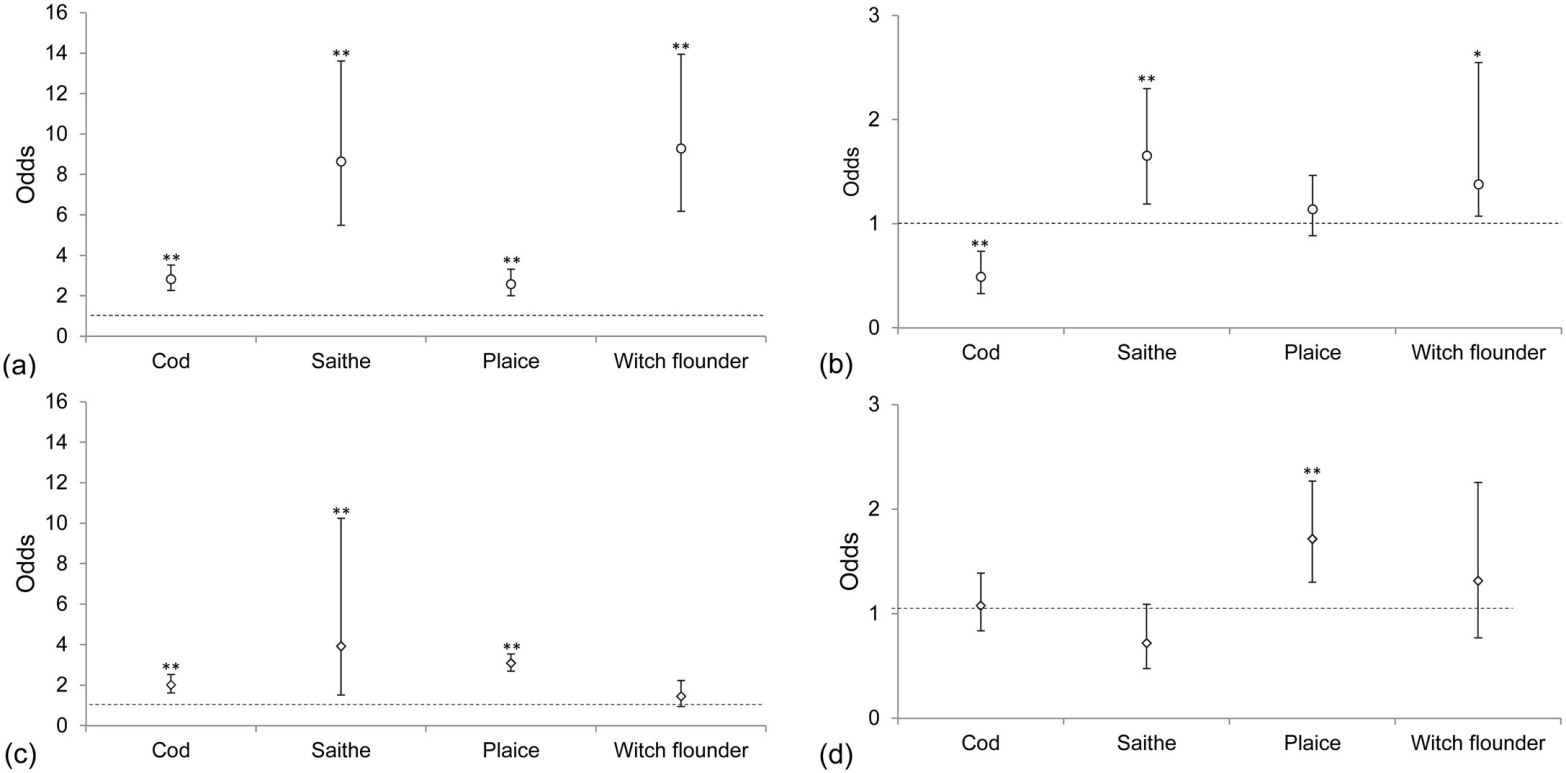
Odds with confidence intervals for obtaining better quality (lower scores) compared with the standard codend for scale loss (○) for newly caught fish from the (a) upper and (b) lower codends of the test gear and discolorations (◇) for newly caught fish from the (c) upper and (d) lower codends of the test gear. The test and standard codends were equal for odds = 1 (dotted line). The results were statistically significant at *p < 0.05 and **p < 0.01 when the confidence intervals did not include 1.

**Fig 6:**
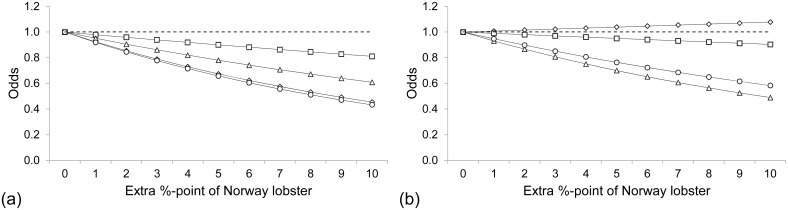
The odds for less scale loss cod (○), saithe (◇), plaice (□), and witch flounder (△) with increasing proportion of Norway lobster relative to fish in the catch of the lower (a) and standard codends (b). When the odds were < 1, the scale loss was higher than that of the starting point (---), whereas for odds > 1 the scale loss was lower.

A total of 4688 newly caught fish (1787 cod, 784 saithe, 1338 plaice, and 779 witch flounder) were assessed by scientists aboard the fishing vessel. Scale loss was the dominant body damage and was exhibited to varying degrees in 76% of all assessed fish (Fig 4). All four fish species caught in the upper codend of the test gear had significantly lower (Wald test, p < 0.01) scores (i.e., had less scale loss) than those caught in the standard codend (Table 3, Fig 5a). The results from the lower codend varied among species. Cod from the lower codend had a higher score and lost significantly (Wald test, p < 0.01) more scales than those caught in the standard codend, whereas the opposite was true for saithe (Wald test, p < 0.01, Fig 5b). Lower scores were given to plaice and witch flounder from the lower codend, but the difference was only significant (Wald test, p < 0.05) for witch flounder. Overall, an increase in the proportion of Norway lobster led to an increased probability of scale loss for fish caught in the lower (likelihood ratio test, p-value: 0.007) and standard (likelihood ratio test, p-value: 0.002) codends. This result was also obtained separately for each species, except for saithe, for which the probability increased with increasing proportion of Norway lobster in the standard codend (Fig 6).

**Fig 7:**
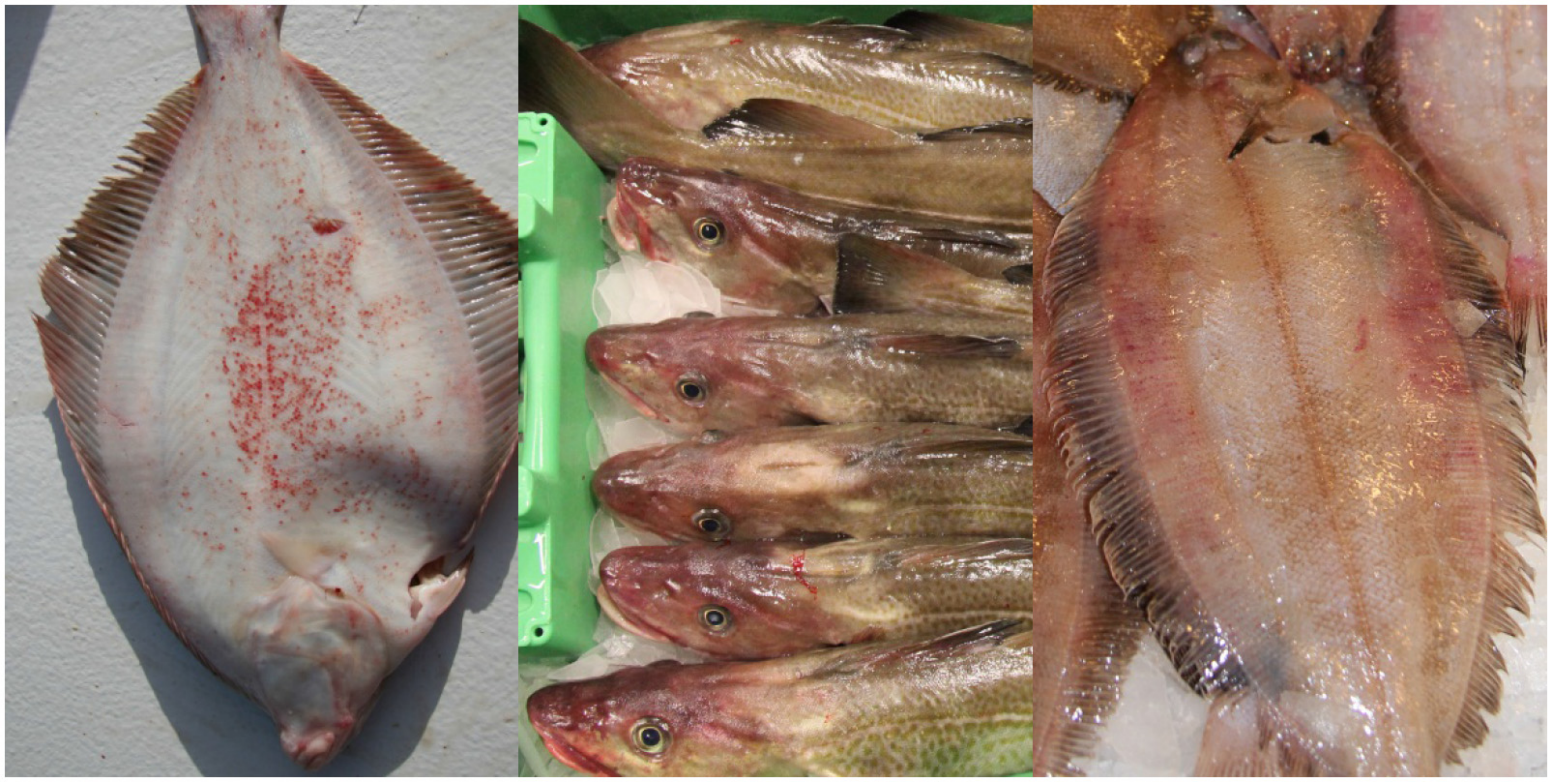
Discolourations: blood spots in plaice (left); bruises on the head of cod (middle); bruises along the body of witch flounder (right).

Discolourations were found in 65% of the assessed individuals. The level of discolouration increased significantly (Wald test, p < 0.01) with an increase in catch weight when all species and codends were pooled. When fish were caught in the upper codend the level of discolouration was significantly lower (Wald test, p < 0.01) for all species except witch flounder compared to those caught in the standard codend (Fig 5c). When comparing fish caught in the lower codend to those caught in the standard codend, the only significant difference was found for plaice, which had significantly less (Wald test, p < 0.01) discolouration when caught in the lower codend (Fig 5d). Of the four fish species, saithe was most resistant to discolouration in the catch process and consistently had the lowest score (Table 3). Plaice and witch flounder had the highest scores, as they suffered both bruises and blood spots. Plaice were most susceptible to blood spots, which were highly visible on the blinded side (Fig 7). Bruises were visible as red discolourations on the head, especially on cod, but they were also found along the body, where they might affect quality of the fillets (Fig 7).

There were no significant differences in the degree of injuries between the three codends for any species (likelihood ratio test, p-value: 0.81). Injuries were found in only 3% of the assessed fish (Table 3).

#### Landed fish

**Fig 8:**
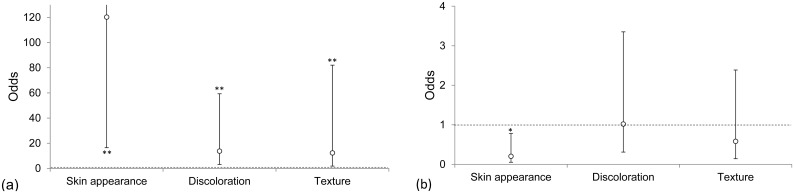
Odds with confidence intervals for obtaining lower quality scores compared with the standard codend for the quality parameters of landed fish caught in the (a) upper and (b) lower codends of the test codend. The test and standard codends were equal for odds = 1 (dotted line). The results were statistically significant at *p < 0.05 and **p < 0.01 when the confidence intervals did not include 1.

Seventy-two samples of landed fish were analysed by the assessment panel (Table 3). Fish caught in the upper codend had significantly better (Wald test, p < 0.01) scores, and thus better perceived quality, for all three quality measures (skin appearance, discolourations, and texture) compared to those caught in the standard codend (Fig 8a). The skin appearance of fish caught in the lower codend was significantly worse than that of fish caught in the standard codend, whereas no significant differences were found with respect to discolourations and texture (Fig 8b). The assessments made by the assessment panel and the Fishermen’s Collection Central did not differ significantly (likelihood ratio test, p-value: 0.19).

#### Fillets

**Fig 9:**
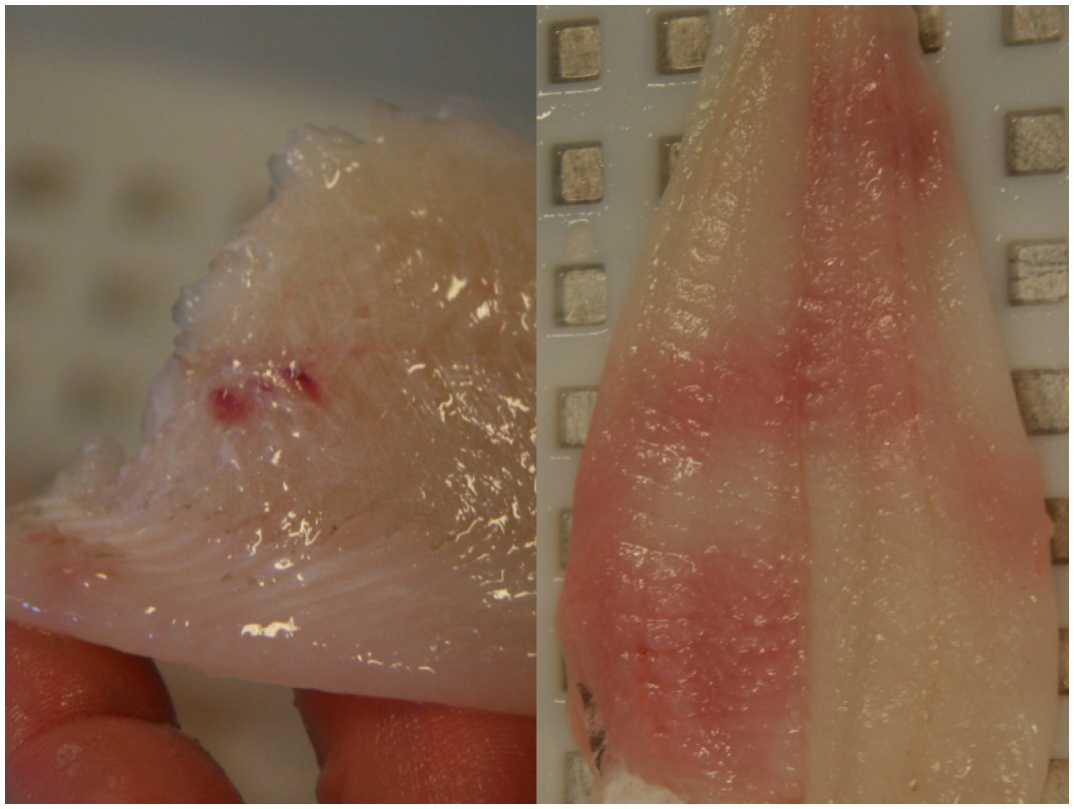
Blood spots (left) and severe degree of bruising (right) in fillets from plaice.

**Fig 10:**
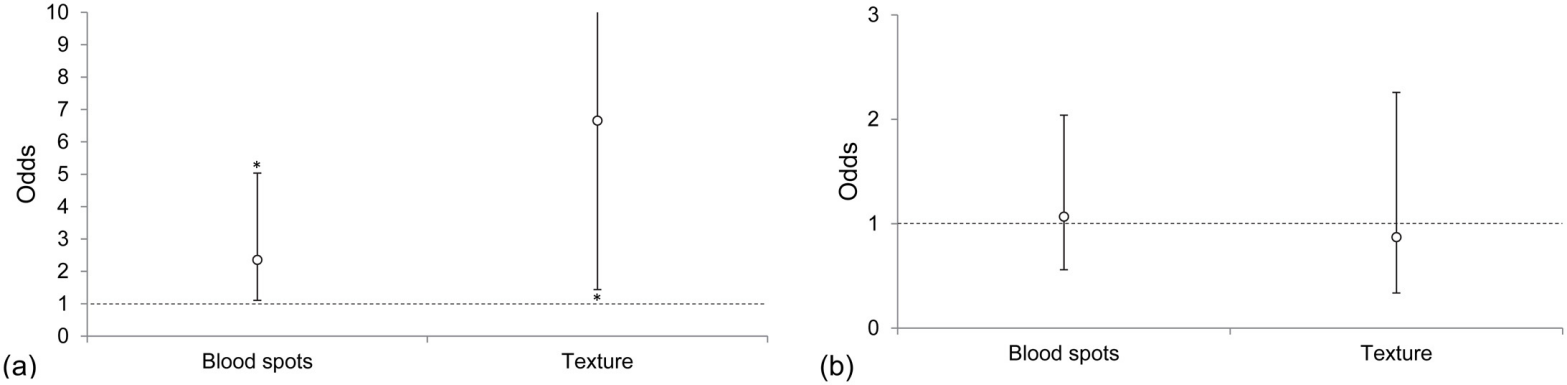
Odds with confidence intervals for obtaining lower quality scores compared with the standard codend for the quality parameters of fillets from fish caught in the (a) upper and (b) lower codends of the vertically divided fishing gear. The test and standard codends were equal for odds = 1 (dotted line). The results were statistically significant at *p < 0.05 when the confidence intervals did not include 1.

The assessment panel assessed 274 fillets and found blood spots in 27% (3% severe) of the sample (Fig 9). Blood spots on fillets from fish caught in the upper codend were significantly less common (Wald test, p < 0.05) compared with fillets from fish caught in the standard codend (Fig 10a). No differences in blood spots were detected between the lower and standard codends (Fig 10b). In these two codends, the score of blood spots in plaice fillets was lower than that in fillets from the other species (Table 3).

The fillet texture of fish from the upper codend was significantly (Wald test, p < 0.05) firmer than that of fish from the standard codend (Fig 10a). However, the texture of fillets of fish caught in the lower and standard codend did not differ significantly (Fig 10b). For both landed fish and fillets, the probability of being assessed as having a lower texture quality increased significantly (Wald test, p-values: 0.04 and 0.0002, respectively) for each day that passed since the day of catch.

**Fig 11:**
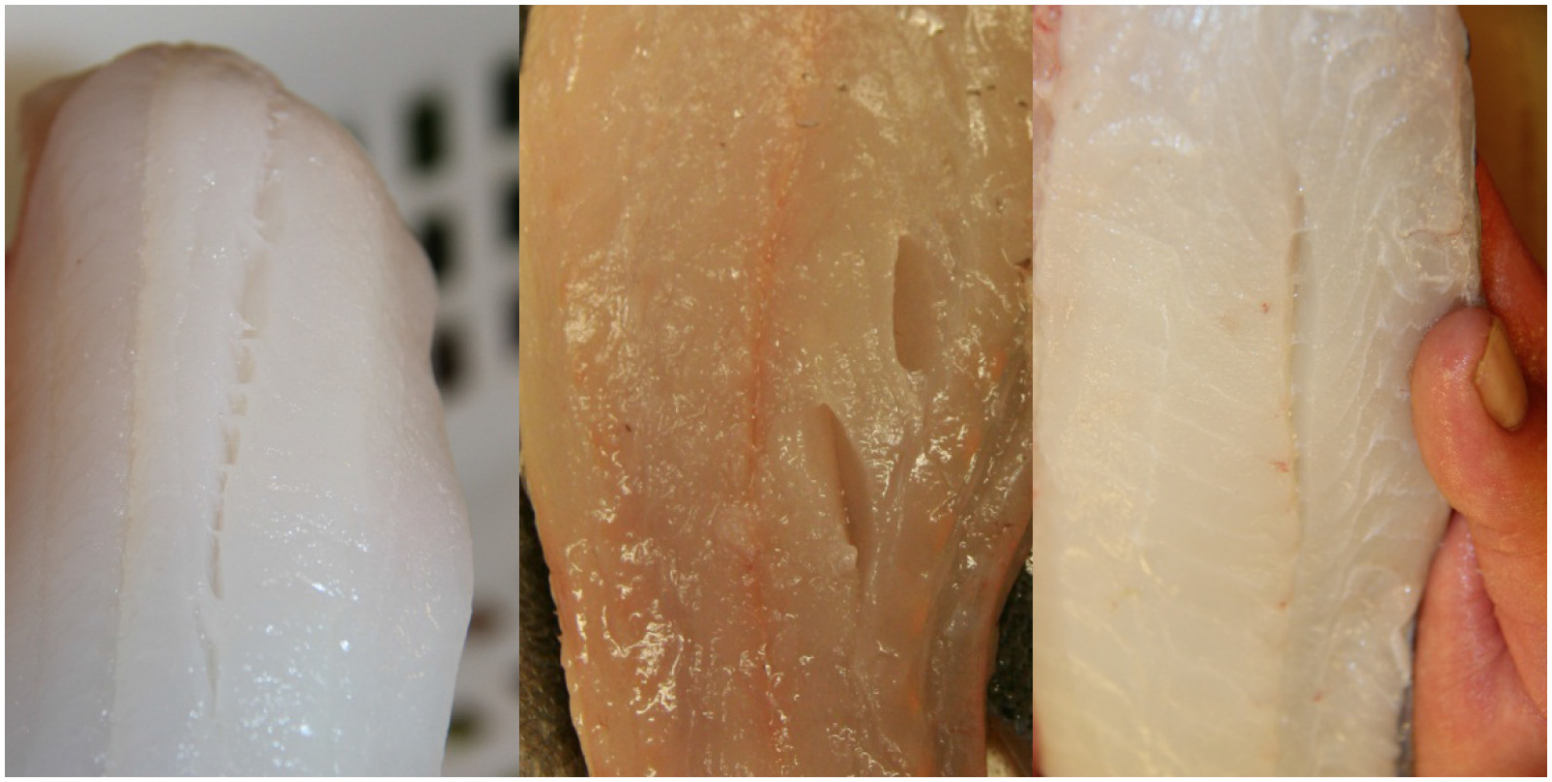
Gaping in cod (left), saithe (middle), and witch flounder (right) fillets.

There were no statistical differences in bruises or gaping between any of the codends (likelihood ratio test, p-value: 0.13 and 0.96, respectively). The assessment panel found bruises in 27% (5% severe) of the assessed fillets, and 48% (19% severe) had gaping. For all codends combined, bruises were most pronounced for cod, saithe and plaice and least pronounced for witch flounder (Fig 9, Table 3), while gaping was most pronounced for cod and least pronounced for plaice (Fig 11, Table 3).

## Discussion

This study demonstrated that relatively simple changes to the design of trawl gear used in the demersal mixed fisheries targeting Norway lobster can increase the quality of fish in the three assessed steps in the value chain (i.e., newly caught fish, landed fish, and fillets). The assessments made by scientists and the assessment panel were similar for whole fish. The horizontally separated codend tested in the commercial fishery significantly improved the quality of all four fish species compared with the standard fishing gear that is used today. This difference was mainly due to improvements in the quality parameters scale loss and skin appearance, discolourations, and texture of fish caught in the upper codend of the test codend. The overall quality of the catch from the lower codend was similar to that of the catch from the standard codend. Different mechanisms at work during the fishing process, such as catch composition, gear selection, and stress, may be responsible for the observed improvements in quality parameters, and the effect of each quality parameter on the fish price may vary depending on the specific end-product for which the fish is intended.

### Newly caught fish

Severe scale loss may restrict sale of fish to customers that present whole fish for their consumers. As expected, separation of fish and Norway lobster into an upper and lower codend during the fishing process resulted in significantly less scale loss in newly caught fish than when the catch was mixed in the standard codend. The lowest level of scale loss was found for fish caught in the upper codend, where the proportion of Norway lobster was lowest and contact with other animals and debris with hard surfaces was limited. The opposite was true for fish caught in the lower codend. The significant increase in scale loss with an increase in the proportion of Norway lobster in the catch for both the lower and standard codends indicated that physical contact between Norway lobster and fish was a major cause of scale loss and dull body surface in fish. In addition to Norway lobster, substantial amounts of other organisms with hard and spiny surfaces, such as sea stars and sea urchins, and debris were caught in the lower and standard codends. These are also likely to abrade the skin of fish during fishing and handling. Species-specific differences in scale loss were detected, as saithe and witch flounder were more prone to scale loss than cod and plaice. The significant improvement in scale loss recorded by scientists was also noted by the assessment panel during their assessment of skin appearance of landed fish. In addition, the assessment panel commented on the glossy look of fish caught in the upper codend.

It is possible that different types of netting in the fishing gear may have different effects on the quality of the fish skin, as fish can rub against the net in escape attempts or lie pressed against it due to water flowing through the gear can lose their scales [37]. Fish may scrape against knotted netting [7] or be affected by the type of mesh. For example, square meshes have tension only in the longitudinal mesh bars during the fishing process whereas diamond meshes have tension in all four mesh bars [38]. However, results from the current study suggest that the netting material had little effect on damage to fish. Similar results were obtained from the lower codend, which was made of knotless Ultracross netting, and the standard codend, which was constructed of knotted PET-netting. Digre et al. [7] also did not detect significant differences between fish caught in a diamond mesh codend with those caught in knotted polyamide netting and a T90 (diamond meshes turned 90 degrees) codend with knotless netting in the aft part.

Based on assessments by both scientists and the assessment panel, fish caught in the upper codend suffered significantly less discolouration compared with fish caught in the other two codends. Few animals with hard outer surfaces that lead to blood spots were caught in the upper codend, and the catch weight in this part of the codend was reduced, which likely decreased the amount of bruising. The level of discolouration significantly increased with increasing catch weight. However, because the catch was collected in two codends, the weight exerted on each individual was probably less than in the standard codend in which the total catch was mixed. Furthermore, the total catch weight from the vertically separated gear was lower than that from the standard gear because of the more open square meshes and larger meshes in the upper codend that made it possible for small individuals to escape from the gear. Positive relationships were found between bruises and catch size in other studies with catch sizes of < 5 tonnes [7, 9]. Olsen et al. [10] did not find such a relationship for large haul sizes with haul durations similar to those of the current study. They observed bruises (extravasations) in 20% of their total sample, which was the same as that reported in Digre et al. [7] at smaller catch sizes with similar haul durations (Table 4). A higher proportion (65%) of bruises was found in the current study at catch sizes similar to those reported in Digre et al. [7]. As much as 80% of the assessed fish had bruises in a haul with 3.2 tonnes of cod and lasted for 1.25 hrs [8]. These findings indicate that factors other than catch weight affect the amount of bruising in the catch. The category ‘discolouration’ in the current study included both bruises and small blood stains on the skin. Flatfish were susceptible to small blood stains, which were highly visible on their blind side, and this resulted in higher rates of discolouration than those found in roundfish. Cod sustained more bruises than saithe, although the dark bruises were harder to detect on the dark skin of saithe and thus it is possible that some were missed during assessment.

**Table 4:** Conditions for different studies for which the proportion of individuals with bruises was compared.

	Current study	Digre et al. (2010)	Olsen et al. (2013)	Rotabakk et al. (2011)
Number of individuals assessed	4688	1001	600	20
Number of hauls	9	16	10	1
Codend catch weight (tons); range (mean)	0.1–0.8 (0.4)	0.5–2.8 (1.5)	8.4–21.6 (15.8)	3.2*
Haul duration (hrs); range (mean)	2.4–6.5 (5.3)	2.5–6.0 (4.9)	3.5–7.0 (5.1)	1.25
Fishing depth (m); range (mean)	75–198 (126)	238–370 (384)	190–240 (210)	250–350 (300)

*3.2 tons of cod were caught in this study, but it was not reported whether additional species were caught, which would add to the total catch weight.

### Landed fish

The assessment panel assessed the texture of landed fish to be best when the fish had been caught in the upper codend, while the texture of fish caught in the other two codends was similar. This difference likely was caused by a delay in the time until onset and release of *rigor mortis* for fish caught in the upper codend. Fish that are stressed immediately before they are killed enter *rigor mortis* earlier than fish that are less stressed and their muscles also get softer faster [21, 23, 25, 26, 39]. Fish caught in the upper codend may have experienced less stress compared with those caught in the other codends. The fishing gears were towed simultaneously by the same vessel and their haul duration was identical. Thus, the lower levels of damage to fish caught in the upper codend may be a consequence of lower catch levels in the upper codend and the resulting lower level of stress to the fish. The lower catch levels were caused by higher selectivity of small, unmarketable fish due to the more open square mesh netting in the test codend compared with the diamond meshes in the standard codend. Increasing haul size has previously been shown to increase the stress level and degree of damage to fish [7, 9, 10].

The assessment panel referred to fish caught in the upper codend as newer than those caught in the two other codends even though the fish were caught during the same haul. This difference in perceived freshness was mainly due to the firmer texture of fish from the upper codend. Freshness is coupled to age and shelf life, and lower freshness indicates older fish and shorter remaining shelf life [2, 9, 40]. Differences in perceived freshness can thus have consequences for the price obtained at the fish auction. In the opinion of the assessment panel, the quality of the catch from the upper codend was comparable to that of catches from the Danish seine fishery, which is the catching method known to deliver the best quality among the towed fishing gears. A similar increase in quality of trawled fish can be achieved by keeping fish alive in water tanks aboard the vessel until bleeding and gutting, which reduces discolouration of fillets [10]. The simple changes in the design of the vertically divided fishing gear also resulted in a decrease in the amount of discolouration of whole fish caught in the upper codend.

Separating the catch into different codends and increasing the size selectivity during fishing not only reduced the sorting time aboard the vessel and the time until gutting, washing, and cooling, but it also reduced the catch weight and the pressure exerted on the fish when being hauled aboard the vessel and stored in the holding bin. This improvement in quality may increase the proportion of the catch that can be sold as high value products.

### Fillets

The significantly better quality in texture perceived for landed fish from the upper codend prevailed in fillets. In the opinion of the assessment panel, fish from the upper codend produced a higher proportion of fillets in the pre-rigor state or with firmer texture than those caught in the standard codend. This is in line with results for fillets from unstressed fish [25]. Delay in onset of *rigor mortis* provides the opportunity of pre-rigor filleting, which is economically favourable due to the fast spoilage of fish [41–43]. Fillets produced pre-rigor change shape and become shorter and thicker, but fillet yield and weight loss remain unaffected [44]. The exporting companies that participated in this study usually perform filleting after the onset of rigor mortis because in their experience machine skinning of soft pre-rigor fillets damage the fillets. Indeed, if fillets are from fish that are exhausted from catching and handling, low muscle pH following anaerobic metabolism weakens the strength of muscle connective tissue [26, 45]. Thus, pre-rigor handling of fillets from stressed fish probably leads to more tears and gaps compared to those of fillets from unstressed fish. However, in the current study the representatives of the exporting companies did not perceive significant differences in fillet gaping between any of the codends. As Digre et al. [7] also reported, the level of gaping was generally low, which suggests that economic loss due to pre-rigor filleting may be limited. Even though the fillets became significantly softer for each day that passed since the day of capture (max. 7 days), the degree of gaping was the same. Fillets with pronounced gaping, as seen in fillets from well-fed fish in captivity, are usually not sold as fillet-products and can lead to substantial economic loss for the processing plants [46]. The feeding status was not known for the trawl-caught fish assessed in the current study, but it can be argued that their glycogen stores probably were small compared to fish fed in captivity, resulting in stronger connective tissue (due to higher muscle pH) and thus a low degree of gaping [7, 22, 45].

No differences in the level of bruises were found between the fishing gears. Most bruises were found on the head of the fish, but the head was removed during processing. The significantly lower degree of blood spots for fillets from fish caught in the upper codend was due to the low amount of Norway lobster and other species with spiny surfaces caught in this part of the gear. The levels of discolouration (bruises and blood spots) were 57% and 66% for cod caught in the upper and standard codend, respectively. Rotabakk et al. [8] reported that bruises, which were observed in 80% of the assessed cod, were a major catch-related damage. The level of blood spots for cod fillets was similar to that reported by Digre et al. [7] (30% vs. 33%), but it is unclear whether the category ‘blood spots’ in Digre et al. [7] also included bruises. According to the quality representatives of the exporting companies, removal of fewer blood stains during trimming of fillets increases the fillet yield and the economic value of the fillets (see also [11]). In addition, a reduction of discoloured fillets reduces the proportion of the catch that is used for low quality products such as mince or is thrown away.

The mixed fishery targeting Norway lobster is one of the most important European fisheries, and the annual catches of fish are worth approximately €80 million for the Danish fisheries alone. The current study has shown that relatively simple and cheap solutions can lead to significant improvements in seafood quality that potentially can increase the catch value in nationally important fisheries. There is an increased demand for fresh products and with it comes more stringent product quality requirements. Improvements in the catch handling that reduce stress and damage to fish and that can lead to faster killing are in line with the increased focus on fish welfare among consumers. This may improve the reputation of trawl-caught fish and can make catches from the mixed trawl fishery more attractive to the market [11]. The test codend used in the current study is not legal according to the current technical legislation due to the small mesh size in the lower codend, but it could be legalized by replacing the netting of 60 mm mesh size with 90 mm in the lower codend. This increase in mesh size is however likely to result in loss of valuable Norway lobster [47]. One of the aspects discussed in relation to the discard ban introduced in the European fisheries is to allow less restrictive gear choice in fully monitored fisheries. In such a setting, our test codend would be a suitable choice for the fisheries targeting Norway lobster and mesh sizes in the upper and lower codends could freely be adjusted to the vessels’ real-time fishing situation.

## Supporting Information

S1 Appendix Cumulative logit models with proportional odds. Statistical analysis of the data obtained from the quality assessments.
(DOCX)
