# Supplementary material for: From Fishing to Fish Processing: Separation of Fish from Crustaceans in the Norway Lobster-Directed Multispecies Trawl Fishery Improves Seafood Quality
Source: PLoS One. 2015 Nov 16;10(11):e0140864. doi: 10.1371/journal.pone.0140864 (PMC4646656; doi:10.1371/journal.pone.0140864)
Supplement: S1 Appendix — Statistical analysis of the data obtained from the quality assessments. (DOCX) [file pone.0140864.s001.docx]

# S1 Appendix. Cumulative logit models with proportional odds

When categorical data have an ordering on the response categories, this ordering can be taken into account by the logits of the cumulative probabilities. Let $Y_{i}$ be the response category of the $i$^th^ observation. We model the logit of the cumulative probability of $Y$ falling at or below the $j$^th^ category as

$$logit[P(Y_{i}\leq j)]=log\left( \frac{P(Y_{i}\leq j)}{1-P(Y_{i}\leq j)} \right)=\alpha_{j}+\beta x_{i}, j=1,\ldots,J-1$$

where $\beta$ is a row vector of parameters, $x_{i}$ is a column vector of explanatory variables, $\alpha_{j}$ is a category specific intercept, and $J$ is the number of scale categories. Naturally $P(Y_{i}\leq J)=1$.

From the logit form, the cumulative probabilities themselves are obtained by

$$P(Y_{i}\leq j)=exp(\alpha_{j}+\beta x_{i})/(1+exp(\alpha_{j}+\beta x_{i}))$$

and the category probabilities are calculated recursively by

$$P(Y_{i}=j)=P(Y_{i}\leq j)-P(Y_{i}\leq j-1)$$

with $P(Y_{i}=1)=P(Y_{i}\leq1)$.

A random effect, $U_{i}\sim N(0,\sigma^{2})$, is added to the model as

$$logit[P(Y_{i}\leq j)]=\alpha_{j}+\beta x_{i}+U_{i}, j=1,\ldots,J-1$$
